# Exercise Endurance Effects of Food‐Derived Polysaccharides in Animal Models of Exercise‐Induced Fatigue: A Systematic Review and Meta‐Analysis

**DOI:** 10.1002/fsn3.72529

**Published:** 2026-10-29

**Authors:** Yingxi Zhao, Tianle Zhang, Zixin Wang, Xin Huang, Jiazhou Li, Lijuan Zou, Zheng Li

**Affiliations:** ^1^ Faculty of Chinese Medicine Macau University of Science and Technology Taipa Macao China; ^2^ School of Sports and Health Guangzhou University of Chinese Medicine Guangzhou China; ^3^ Guangzhou University of Chinese Medicine Guangzhou China; ^4^ Clinical Medical College of Acupuncture, Moxibustion and Rehabilitation Guangzhou University of Chinese Medicine Guangzhou China; ^5^ Guangdong Chaozhou Health Vocational College Chaozhou China; ^6^ Guangdong Food and Drug Vocational College Guangzhou China

**Keywords:** exercise fatigue, food‐derived polysaccharide, functional food

## Abstract

The effects of food‐derived polysaccharides on endurance may depend on their source materials, extraction and purification procedures, molecular characteristics, and mode of administration; however, their average effects and transferability across animal models remain uncertain. To quantify effects on exercise endurance and to evaluate how chemical/process characterization, biochemical response domains, and evidence quality support functional‐food interpretation. Controlled animal studies were systematically reviewed; 31 independent StudyIDs from 30 reports were synthesized using random‐effects response ratios (RoM) with Hartung–Knapp intervals. Chemical and processing descriptors and selected biochemical outcomes were mapped as structured evidence layers. Endurance increased overall (RoM 1.707, 95% CI 1.540–1.892; *I* ^2^ = 95.4%), while the 95% prediction interval (0.999–2.917) included the null. Characterization was incomplete: purity/content, molecular weight, monosaccharide composition, and extraction or purification information were recoverable for only subsets of StudyIDs. Biochemical observations clustered around energy‐substrate, lactate/BUN, oxidative‐stress, muscle‐injury, mitochondrial/AMPK, and gut‐microbiota domains, but few comparable records were suitable for effect‐size calculation. Food‐derived polysaccharides show a positive average preclinical endurance signal, but very high heterogeneity, incomplete chemical/process reporting, evidence asymmetry, and sparse harmonized biochemical data constrain claims of efficacy for formulated functional foods.

## Graphical abstract

Across 31 independent animal experiments, food‐derived polysaccharides showed a positive average endurance signal (response ratio 1.707; 95% confidence interval 1.540–1.892). However, marked between‐study heterogeneity, incomplete chemical and process characterization, and sparse comparable mechanistic data limit transferability and do not support a uniform class effect or a functional‐food efficacy claim.

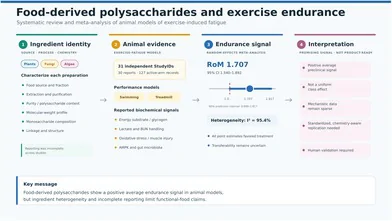

## 1 Introduction

Exercise‐induced fatigue reflects a progressive loss of the capacity to sustain a required level of force, power, or exercise performance. In experimental settings, prolonged or exhaustive exercise is accompanied by depletion of energy substrates, accumulation of fatigue‐related metabolites, oxidative stress, tissue perturbation, and central or peripheral neuromuscular changes (Luo et al. 2019; Twomey et al. 2017). From a food‐biochemistry perspective, candidate interventions should therefore be evaluated not only by performance endpoints but also by the biochemical states that connect food‐derived molecules to energy metabolism, metabolite handling, redox balance, and tissue protection.

Food‐derived polysaccharides occupy a distinctive position at the interface of food chemistry, processing, and functional physiology. Preparations from edible plants, fruits, cereals, algae, mushrooms, fermented foods, and food‐processing by‐products are chemically diverse rather than a single interchangeable class. Their biological activity can vary with molecular weight, monosaccharide composition, glycosidic linkage, branching and conformation, purity, residual protein, extraction conditions, purification strategy, and subsequent chemical or physical modification (Du et al. 2022).

Animal experiments have reported longer exhaustive swimming or treadmill performance after supplementation with polysaccharides from several food‐related sources (An and Luo 2013; Chang et al. 2022; Chen et al. 2014; Hao 2014; Klosterhoff et al. 2018; Li et al. 2020; Xu et al. 2013; Zhao et al. 2021; Zhong et al. 2019; Zhou et al. 2024; Zhu et al. 2020). Two recent reviews summarized source classes, doses, experimental models, structural features, gut‐muscle signaling, and proposed anti‐fatigue mechanisms (Lei et al. 2024; Liu et al. 2024). The remaining gap is food‐biochemical as well as quantitative: it is unclear how a chemically heterogeneous ingredient class maps onto biochemical response domains and functional endurance outcomes across independent animal models.

We therefore conducted a systematic review and meta‐analysis of food‐derived polysaccharides in animal models of exercise‐induced fatigue. The main manuscript focuses on four connected evidence layers: chemical/process characterization, biochemical response, endurance effect magnitude and transferability, and evidence gaps for functional‐food translation. The Supporting Information describes how independent experiments were assigned study IDs (StudyIDs), how database records and intervention arms were reconciled, and how source information was checked.

## 2 Materials and Methods

### 2.1 Reporting Framework and Protocol Status

This review was reported in line with the Preferred Reporting Items for Systematic Reviews and Meta‐Analyses (PRISMA) 2020 statement (Page et al. 2021). Eligibility criteria and eligible sampling‐time windows were defined before screening began. Report‐family linkage, multi‐arm handling, selection among multiple eligible sampling times, and effect‐size construction were specified before data extraction; linked reports were consolidated at the study level before extraction. After data extraction, effect sizes and analyses were completed according to these prespecified rules. The review was not prospectively registered. Detailed database‐reconstruction notes and post hoc audit procedures are provided in the Supporting Information rather than expanded in the main text.

### 2.2 Information Sources and Search Approach

The core search covered PubMed/MEDLINE, Embase, Web of Science, CNKI, Wanfang, and VIP/CQVIP. ClinicalKey was used solely for full‐text retrieval and citation verification and was not included in the database search counts. The executed search strategies, dates, and count evidence are provided in Table S1 and the accompanying search‐strategy workbook.

Across the six core databases, 4412 exported records entered the reconstruction workflow, and 50 additional records from an earlier Wanfang export were added before deduplication. Platform/export discrepancies and source‐specific reconciliation steps are reported in the Supporting Information; in the main text, these records define the screening denominator and do not add separate evidence.

### 2.3 Eligibility Criteria

Eligible studies were controlled animal experiments that evaluated a polysaccharide intervention from an edible or food‐relevant source in a model of exercise‐induced fatigue, endurance, or exhaustion. Report‐level evidence class was separated from quantitative‐synthesis eligibility. Detailed report‐level eligibility classifications are provided in Tables S2 and S3. Each active arm in the primary‐analysis dataset (STRICT_PRIMARY) had to represent an attributable single‐source food‐derived polysaccharide, a nonlethal performance endpoint based on endurance or exhaustion time, and usable group size, mean, and interpretable variance. When several eligible source‐specific active arms shared one control, they were combined only at the StudyID level so that the same control was not reused. Preparations explicitly described as crude, low‐purity, or high‐protein were assigned to a separate purity/composition boundary stratum. Modified products, formulations, non‐attributable mixtures, incompatible death/survival endpoints, companion reports, and reports without usable numerical endurance summaries were assigned to explicit non‐primary classes.

The food‐source boundary was operationalized with a review‐specific two‐tier rule (Table S3A). Tier A (STRICT FOOD‐SOURCE) required the report or source record to present the intervention source explicitly or unambiguously as a conventional food, edible food part or ingredient, edible fruit, vegetable or cereal, edible mushroom or alga, fermented‐food source, or food‐processing by‐product. SOURCE‐B was reserved for food‐medicine boundary materials that were presented mainly as medicinal or tonic sources, or for which conventional dietary‐food status could not be established from the report without importing an external ethnobotanical or regulatory judgment. This rule defines review scope; it does not claim that SOURCE‐B materials are legally non‐food or are never consumed. Source category was assigned at the StudyID/intervention level when a report contained independent cohorts from different sources.

Source scope and preparation purity were handled as separate eligibility dimensions. The 17‐report purity/composition boundary stratum was retained to document non‐strict preparation or attribution boundaries. Only three legacy reports had previously contributed to the primary quantitative set and were eligible for the targeted three‐report legacy reclassification sensitivity analysis (*k* = 34); this was not an all‐17 boundary‐study meta‐analysis.

Sedentary controls were not substituted for exercised controls when the intervention effect was estimated in an exercise model. Studies lacking an attributable polysaccharide contrast, a target exercise‐fatigue model, or a usable comparator were excluded from the relevant quantitative synthesis. The quantitative‐synthesis eligibility flow is provided in Table S6; biochemical outcome sampling strata and final pooling decisions are provided in Table S4.

### 2.4 Study Selection and Report‐Family Handling

The screening and family‐linkage workflow is summarized in Figure 1, with database reconstruction, reviewer‐agreement details for the reconstructed layer, and seed/registry reconciliation documented in the Supporting Information. In brief, 3059 deduplicated records were screened, 749 reports were assessed for eligibility after one unretrieved report, and 30 source reports contributed 31 STRICT_PRIMARY StudyIDs to the endurance synthesis.

**FIGURE 1:**
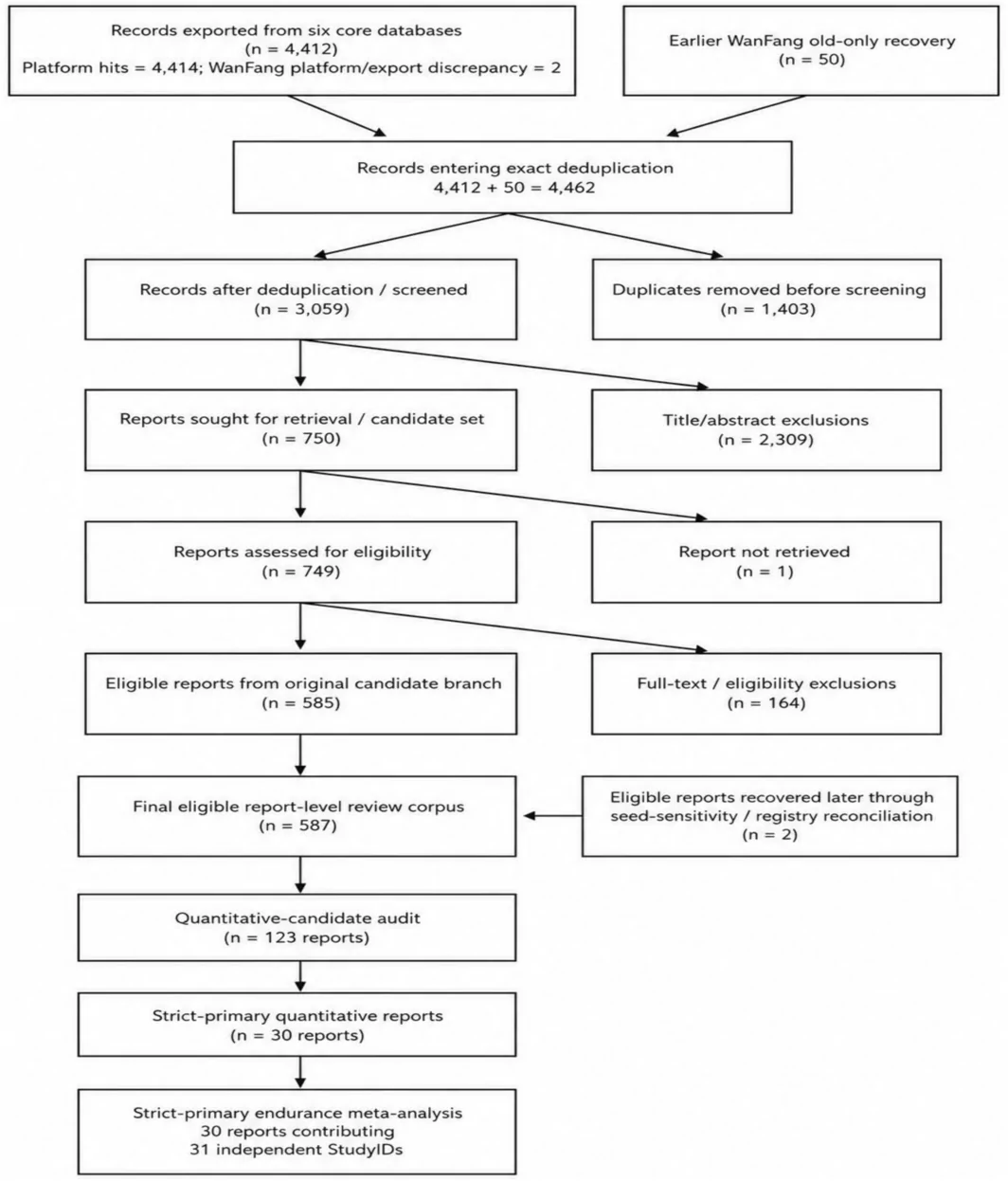
PRISMA 2020 flow diagram. The flow starts from 4412 retained six‐core export rows plus 50 documented earlier Wanfang old‐only records, proceeds through deduplication and screening, and identifies the 30 source reports contributing 31 independent STRICT_PRIMARY StudyIDs. Detailed platform/export reconciliation, seed‐sensitivity recovery, and registry reconciliation are provided in the Supporting Information.

Potential companion reports were linked into study families using the intervention, authorship, institution, publication year, animal design, dose, treatment duration, comparator, and outcome timing. If an identical cohort could not be established, we used conservative deduplication or HOLD rules rather than forcing reports into the same study family.

### 2.5 Data Extraction and Quantitative Unit of Analysis

For each study, we extracted animal and intervention characteristics, exercise model, comparator, treatment duration, outcome timing, group sample size, mean, and dispersion. For food‐biochemical interpretation, we also extracted recoverable source/material, fraction or preparation name, administration route, extraction/preparation method, purification or fraction type, polysaccharide purity/content, molecular weight, monosaccharide composition, structural or linkage information, and protein and uronic acid information, where available. The Supporting Information and reproducibility workbook document study IDs, source records, data extraction from figures, and record reconciliation. Biochemical records suitable for effect‐size calculation are listed in Table S5. StudyID characteristics and effect estimates are provided in Table S7, and the legacy reclassification sensitivity set is documented in Table S6A. NR indicates that information could not be obtained from the available source records; it does not mean the original study did not measure the outcome.

When several eligible active arms shared one control within an independent experiment, the active arms were combined before meta‐analysis using standard shared‐control methods (Higgins et al. 2019):

The combined *n* was the sum of active‐group sample sizes.
The combined mean was the sample‐size‐weighted active‐arm mean.
The combined SD incorporated within‐group variance and between‐arm mean dispersion.

This approach prevented repeated use of a shared control. The primary estimand was the StudyID‐level average effect across all eligible active polysaccharide arms within an independent animal experiment, not the effect of a specific dose. In the shared‐control RPT‐0037 experiment, all eligible source‐specific active arms were combined into one primary StudyID effect; turnip‐only and maca‐only replacements were examined in post hoc shared‐control sensitivity analyses.

After the primary model had been fixed, descriptive layers were generated to support biological interpretation without changing the estimand. These layers included observed endurance summaries, reporting‐completeness counts, a standardized source/fraction chemistry and processing matrix, a biochemical evidence matrix, and a within‐study nominal dose‐pattern audit. Technical audit fields are reported in Supporting Information tables, whereas the main text uses these layers to interpret chemical heterogeneity, biochemical plausibility, and functional‐food translation limits.

### 2.6 Effect Measures and Meta‐Analysis

The primary endurance effect measure was the response ratio (RoM = mean treatment/mean control), analyzed on the natural‐log scale (Hedges et al. 1999). For the endurance outcome, an RoM > 1 favors the polysaccharide intervention. Sampling variance was calculated as:

$$\mathrm{Var}\left(\text{lnRoM}\right)=\mathrm{SD}\_T^{2}/\left(n\_T\times \text{Mean}\_T^{2}\right)+\mathrm{SD}\_C^{2}/\left(n\_C\times \text{Mean}\_C^{2}\right),$$

where *T* and *C* denote treatment and control, respectively.

Statistical analyses were performed in R version 4.4.2 using the metafor, metasens, and ggplot2 packages. Random‐effects models used restricted maximum likelihood estimation of *τ* ^*2*^ and Hartung–Knapp 95% confidence intervals (IntHout et al. 2014). Heterogeneity was summarized with *Q* and *I* ^2^ (Higgins et al. 2003). The 95% prediction interval was calculated on the log‐RoM scale as $\hat{\mu }\pm t_{0.975,k-2}\times \surd \left({\hat{\tau }}^{2}+\mathrm{SE}{\left(\mathrm{HK}\right)}^{2}\right)$, where $\hat{\mu }$ is the pooled log‐RoM, ${\hat{\tau }}^{2}$ is the restricted maximum likelihood estimate of between‐study variance, SE(HK) is the Hartung–Knapp standard error of the pooled mean, and *k* is the number of studies. The limits were then exponentiated to the RoM scale. Individual StudyID effects and synthesis estimates are presented in the forest plot and summary tables.

For biochemical outcomes reported on different scales, Hedges' *g* was oriented so that positive values indicated benefit (Hedges 1981); signs were reversed for biomarkers for which lower values were favorable. Sampling classes were defined before pooling. Immediate or same‐session post‐exhaustion observations were not combined with delayed‐recovery or fixed‐duration exercise samples. Exploratory pooling required at least three compatible studies within a sampling family.

### 2.7 Risk of Bias

Risk of bias was assessed with the 10‐domain Systematic Review Centre for Laboratory animal Experimentation (SYRCLE) tool (Hooijmans et al. 2014). The final 31‐StudyID matrix combines eight StudyIDs assessed in the initial phase with 23 additional StudyIDs independently assessed by two reviewers under the same conservative rules. For these 23 StudyIDs, 230 domain judgments were compared. LOW‐versus‐UNCLEAR disagreements were rechecked against the full text and resolved conservatively as UNCLEAR when an explicit low‐risk safeguard could not be demonstrated; potential HIGH judgments were adjudicated against full‐text evidence. The final 31‐StudyID SYRCLEconsensus matrix is provided in Table S8, and reviewer disagreements and reconciliation are reported in Table S9.

### 2.8 Small‐Study Effects

A funnel plot and Egger‐type regression (Egger et al. 1997) were used only as exploratory diagnostics for the endurance outcome. In the final STRICT_PRIMARY dataset (*k* = 31), the regression was nonsignificant (*p* = 0.844). Because between‐study heterogeneity was very high, this result was not interpreted as evidence that publication bias was absent.

### 2.9 Certainty of Evidence

A formal certainty‐of‐evidence framework was not prespecified, and we did not introduce a post hoc rating after completing the synthesis. The SYRCLE assessment addresses risk of bias within individual studies and was not treated as a substitute for rating certainty across the body of evidence. Instead, evidence strength and limitations were interpreted from the magnitude and precision of pooled estimates, the prediction interval, heterogeneity, sensitivity analyses, reporting‐bias diagnostics, and the SYRCLE risk‐of‐bias assessment.

## 3 Results

### 3.1 Study Selection

Figure 1 summarizes study selection. The reconstruction workflow began with 4462 records entering deduplication, of which 3059 remained for screening; 749 reports were assessed for eligibility after one candidate report could not be retrieved. Detailed StudyID‐level characteristics and quantitative provenance are provided in Table S7, while database and search‐reconstruction documentation is provided in the Supporting Information and accompanying reproducibility materials. The broader eligible corpus contained 587 reports, as profiled in Table S10, but only 30 source reports contributed 31 independent STRICT_PRIMARY StudyIDs to the primary endurance meta‐analysis. Detailed platform/export reconciliation, seed‐sensitivity recovery, and registry reconciliation are reported in the Supporting Information.

The 587‐report corpus was retained as a Supporting Information evidence‐landscape map rather than as the denominator for quantitative synthesis. It shows that the literature contains many qualitative, boundary, mixture/attribution, and reporting‐limited records in addition to the STRICT_PRIMARY studies. The main Results therefore distinguish the broad food‐polysaccharide literature from the smaller set of independent experiments that could support quantitative endurance synthesis.

### 3.2 Characteristics of the Strict Endurance Studies

The STRICT_PRIMARY synthesis contained 31 independent animal experiments from 30 reports and 127 primary‐effect arm records; Table 1 gives the source citation for each StudyID, and Table S7 provides detailed StudyID characteristics and effect estimates. The sum of endpoint‐specific sample sizes across the arm records was 1232, which is a descriptive arm‐level total rather than the effective sample size of the meta‐analysis. Group *n* ranged from 2 to 20, and StudyID‐level RoM values ranged from 1.121 to 3.443. Twenty‐seven StudyIDs involved mice, three involved rats, and the animal species could not be determined for one study from the available source text. Twenty‐five StudyIDs explicitly used swimming exhaustion, two used treadmill/running exhaustion, and four lacked enough detail for finer modality classification. Reported treatment schedules ranged from an acute first‐stage screen tested 15 s after gavage to 7 weeks of supplementation. Broad source categories comprised 15 plant/other, 13 fungal, and three algal StudyIDs. The plant/other category includes sources such as okra, acerola, oats, garlic, apple pomace, green tea, and ginger. Observed StudyID‐level summaries are provided in Table S11, and individual preparations are listed in Table S12.

**TABLE 1:** Characteristics of the 31 independent StudyIDs in the strict‐primary endurance synthesis.

StudyID	Year	Species/strain/sex	Exercise modality	Intervention/preparation	Dose/duration	*n*/group	Exhaustion criterion	References
ST‐APPLEPOMACE‐2022	2022	NR/NR/NR	Exercise exhaustion; detail NR	ACAPP/ALAPP	ACAPP/ALAPP source‐labeled 50/100/200 levels/NR	8	NR	Chang et al. (2022)
ST‐SPIRULINA‐2020	2020	Rat/Sprague–Dawley/Male	Treadmill/running exhaustion	PSP	PSP 50, 100, 200 mg/kg/NR	10	Exhaustion endpoint reported; criterion NR in archived abstract	Zhu et al. (2020)
ST‐HOS‐2020	2020	Mouse/NR/NR	Exhaustive swimming	HOS pectin	HOS low/middle/high; numeric dose NR/NR	10	NR	Li et al. (2020)
ST‐TIP‐2019	2019	Mouse/ICR/NR	Exhaustive swimming	TIP	TIP low/middle/high; numeric dose NR/NR	8	NR	Zhong et al. (2019)
ST‐ACEROLA‐2018	2018	Mouse/NR/NR	Exercise exhaustion; detail NR	ACWS pectin	50, 100, 200 mg/kg/28 days	5	NR	Klosterhoff et al. (2018)
PST‐0045	2014	Mouse/NR/NR	Exhaustive swimming	POP	100, 200, 400 mg/kg/28 days	12	Exhaustion endpoint reported; criterion NR in archived abstract	Chen et al. (2014)
PST‐0148	2024	Mouse/NR/NR	Exhaustive swimming	PYB‐1/PYB‐2	PYB‐1/PYB‐2 low/middle/high; numeric dose NR/NR	18	NR	Zhou et al. (2024)
PST‐0053	2013	Rat/NR/NR	Exercise exhaustion; detail NR	Oat β‐glucan	Oat β‐glucan active arm; numeric dose NR/NR	8	NR	Xu et al. (2013)
ST‐LAMINARIA‐2016	2016	Mouse/Kunming/Male	Exhaustive swimming	Laminaria PS	75, 150, 300 mg/kg/28 days	12	Exhaustive swimming; full‐text endpoint	Yan and Hao (2016)
ST‐HERICIUM‐2015‐ENG	2015	Mouse/ICR/Male	Exhaustive swimming	Hericium PS	50, 100, 200 mg/kg/28 days	10	Exhaustive swimming	Liu et al. (2015)
ST‐CASSAVA‐2013	2013	Rat/Sprague–Dawley/Male	Treadmill/running exhaustion	Cassava PS	500 mg/kg/day/6 days supplementation	10	Treadmill/running exhaustion	Yen et al. (2013)
ST‐GARLIC‐2024	2024	Mouse/ICR/Male	Loaded exhaustive swimming	Garlic GP	1.25 and 2.5 g/kg BW/7 weeks	20 (primary endurance); biochemical *n* = 8; microbiota *n* = 7	Loaded exhaustive swimming	Li et al. (2024)
ST‐APPLE‐PAP1‐2023	2023	Mouse/Kunming/Male	Exhaustive swimming	PAP‐1	50, 100, 200 mg/kg/4 weeks	6 for endurance/biochemical outcomes; body‐weight figure says 10	Exhaustive swimming	Li et al. (2023)
ST‐LENTINUS‐2020	2020	Mouse/Kunming/Male	Exhaustive swimming	Lentinus PS	20, 60, 180 mg/kg/day/NR	10	Exhaustive swimming	Kang and Ren (2020)
ST‐DICTYOPHORA‐2020	2020	Mouse/NR/NR	Loaded exhaustive swimming	Dictyophora PS	100, 200, 300 mg/kg/Source inconsistency: 4 weeks vs. 15 days	5	5% body‐weight load; 8‐s submersion criterion	Wang and Wang (2020)
ST‐DICTYOPHORA‐2016	2016	Mouse/NR/NR	Loaded exhaustive swimming	Dictyophora PS	100, 300 mg/kg/NR	6	5% body‐weight load; 8‐s submersion criterion	Ye et al. (2016)
ST‐TURNIP‐MACA‐2017	2017	Mouse/NR/Mixed (5 male +5 female in endurance subset)	Loaded exhaustive swimming	Turnip + maca PS	Turnip 25/50/100 + maca 25/50/100 mg/kg/NR	10 (target loaded‐swim subset)	Loaded exhaustive swimming; shared control	Tang (2017)
ST‐MACA‐2015‐CHEN	2015	Mouse/NR/NR	Exhaustive swimming	Maca PS	Maca low/middle/high/NR	15 reported in thesis; analytic endurance *n* = 6 per companion	Independent maca cohort	Chen (2015)
ST‐KELP‐2015‐CHEN	2015	Mouse/NR/NR	Exhaustive swimming	Kelp PS	Kelp low/middle/high/NR	15	Independent kelp cohort	Chen (2015)
ST‐HERICIUM‐2015‐ZHANG	2015	Mouse/NR/NR	Loaded exhaustive swimming	Purified Hericium fraction	3 and 9 mg/20 g BW/NR	12 allocated; endurance table *n* = 10	Loaded exhaustive swimming	Zhang (2015)
ST‐LONGAN‐2017	2017	Mouse/NR/Male	Loaded exhaustive swimming	Longan PS	100, 200, 1400 mg/kg/Source inconsistency: 7 vs. 30 days	12 allocated; endurance table *n* = 10	5% body‐weight load; 10‐s submersion criterion	Nie et al. (2017)
ST‐HERICIUM‐2015‐YANG	2015	Mouse/Kunming/Male	Loaded exhaustive swimming	HEPN/HEPA	Purified HEPN + HEPA/15 days	10	Loaded exhaustive swimming	Yang et al. (2015)
ST‐PLEUROTUS‐POPH2	2020	Mouse/Kunming/Male	Loaded exhaustive swimming	POPH‐2	50, 100, 200 mg/kg/NR	10	Loaded exhaustive swimming	Liu (2020)
ST‐GREENTEA‐TPS41	2025	Mouse/C57BL/6/Male	Exhaustive swimming	TPS4‐1	30, 100 mg/kg/NR	10 (50 mice/5 groups)	Exhaustive swimming	Zhou et al. (2025)
ST‐AURICULARIA‐AAPIIIA	2008	Mouse/NR/NR	Exhaustive swimming	AAPIII‐a	AAPIII‐a 50, 100, 150 mg/(kg bw·day)/21 days	10 randomly selected for endurance	Forced/loaded exhaustive swimming	Zhu (2008)
ST‐MORCHELLA‐FRACTION‐SCREEN	2020	Mouse/ICR/Male	Loaded exhaustive swimming	Morchella fractions (10)	10 first‐stage fractions, 2 mg/mL, 1 mL/Acute; test 15 s after gavage	2/group in strict first‐stage screen	Loaded exhaustive swimming; all 10 first‐stage fractions retained	Liang et al. (2020)
ST‐AGARICUS‐ABP1	Online first/NR	Mouse/Kunming/Male	Exhaustive swimming	ABP‐1	10, 20, 40 mg/kg/NR	12	Exhaustive swimming	Ding et al. (2026)
ST‐MACA‐MPS	2019	Mouse/Kunming/NR	Loaded exhaustive swimming	MPS	20, 100 mg/kg/30 days	20 allocated; 10 preassigned to endurance	Loaded exhaustive swimming	Li et al. (2019)
ST‐TREMELLA‐TFP	2024	Mouse/C57BL/6/Male	Exhaustive swimming	TFP	50, 100, 200 mg/kg/2 weeks	10	Exhaustive swimming	Huang et al. (2024)
ST‐ARMILLARIA‐AGP	2023	Mouse/C57BL/6J/NR	Exercise exhaustion; detail NR	AGP	AGP purified polysaccharide/NR	10	Weight‐bearing/exhaustive swimming	Sun (2023)
ST‐GINGER‐ZOPA1	2024	Mouse/Kunming/Male	Exhaustive swimming	ZOPA‐1	ZOPA‐1 low/high/NR	20	Exhaustive swimming	Jing et al. (2024)

*Note:* The Reference column uses Author and year citations. The intervention/preparation column uses concise standardized labels; detailed biological source, fraction/preparation, extraction/purification, and chemistry information are provided in Table S12. The two independent RPT‐0183 cohorts are retained as distinct StudyIDs.

Abbreviations: *n*, animals per group; NR, not reported or not recoverable from the available source material; PS, polysaccharide.

Several study descriptors that are important for biological transportability were incompletely reported. Strain was recoverable for 17/31 StudyIDs (54.8%), sex for 16/31 (51.6%), treatment duration for 14/31 (45.2%), and an exhaustion criterion or comparable model detail for 23/31 (74.2%). In Table 1, ten experiments are described as loaded swimming; three specify a 5% body‐weight load and an 8‐ or 10‐s submersion criterion. Administration route was recoverable for only 7/31 StudyIDs (22.6%), mainly gavage/intragastric or direct oral administration rather than verified incorporation into a conventional food matrix. Detailed observed‐versus‐synthetic active summaries and StudyID audit flags are retained in Tables S11 and S12.

### 3.3 Chemical and Processing Characterization of Polysaccharide Preparations

Chemical/process characterization was treated as a primary Results layer because food‐derived polysaccharides are not interchangeable ingredients. Table S12 separates biological source/material, fraction/preparation name, extraction/preparation method, purification/fraction type, purity/content, molecular‐weight information, monosaccharide composition, structural/linkage notes, and protein and uronic acid information, where available.

Coverage was incomplete. Quantitative purity/content was recoverable for 5/31 StudyIDs (16.1%), molecular weight for 8/31 (25.8%), monosaccharide composition for 8/31 (25.8%), extraction or preparation method for 15/31 (48.4%), and structural, linkage, or fraction information for 12/31 (38.7%). Characterized examples included okra stem pectin, *Tuber indicum* polysaccharide, acerola ACWS pectin, purified oat beta‐glucan, garlic GP, apple‐pomace PAP‐1, green‐tea TPS4‐1, Armillaria AGP, and ginger ZOPA‐1 (Klosterhoff et al. 2018; Li et al. 2020, 2024; Xu et al. 2013; Zhong et al. 2019; Huang et al. 2024; Jing et al. 2024; Sun 2023; Zhou et al. 2025). These data permit interpretation in light of the reported chemical characteristics but are too sparse for a structure–activity meta‐regression. In incompletely characterized preparations, residual proteins or other co‐extracted components also limit attribution specifically to polysaccharides.

### 3.4 Primary Endurance Meta‐Analysis

The final analysis included 31 independent StudyIDs. On average, food‐derived polysaccharide supplementation was associated with longer endurance (RoM 1.707, Hartung–Knapp 95% CI 1.540–1.892; *τ* ^*2*^ = 0.066). Heterogeneity was very high (*I* ^2^ = 95.4%; *Q* = 645.4, df = 30), showing substantial variation in effect magnitude across chemically diverse preparations and experimental settings. Figure 2 presents the StudyID estimates, random‐effects weights, pooled estimate, and prediction interval; Table 2 summarizes the primary meta‐analysis and the robustness and heterogeneity analyses.

**FIGURE 2:**
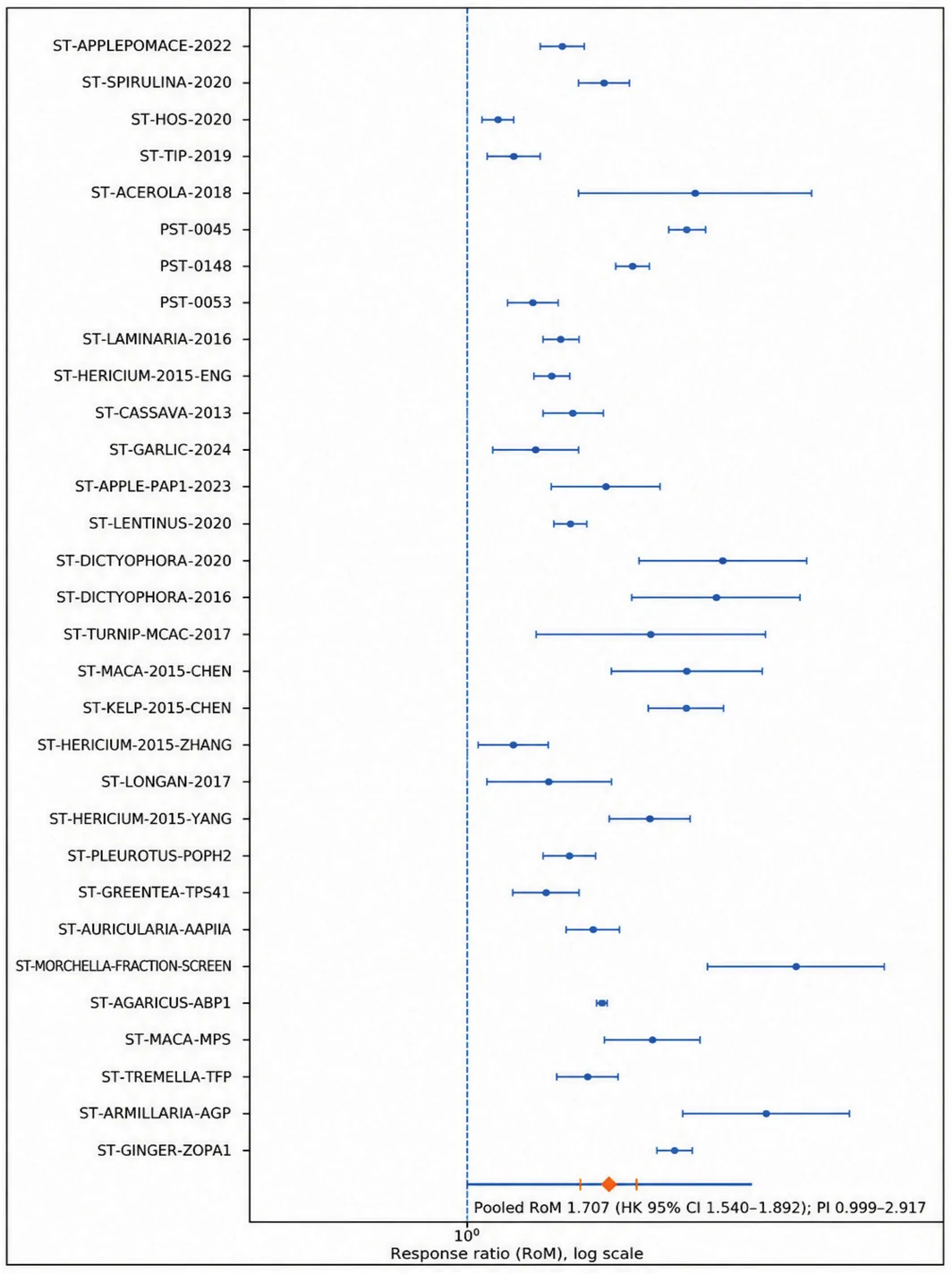
Forest plot of the 31 independent StudyID‐level response ratios. StudyID labels distinguish independent cohorts, including the two cohorts from RPT‐0183. The pooled restricted maximum likelihood/Hartung–Knapp estimate is RoM 1.707 (95% CI 1.540–1.892), and the 95% prediction interval is 0.999–2.917, which includes the null.

**TABLE 2:** Primary endurance meta‐analysis and robustness/heterogeneity analyses.

Analysis	*k*	Pooled RoM	Hartung–Knapp 95% CI	*I* ^*2*^ (%)	95% prediction interval
Strict‐primary endurance synthesis	31	1.707	1.540–1.892	95.4	0.999–2.917
Exclude StudyIDs with ≥ 1 HIGH SYRCLE domain	26	1.721	1.532–1.934	95.9	0.979–3.026
Source category: Plant/other	15	1.652	1.438–1.897	96.8	0.976–2.795
Source category: Fungal	13	1.776	1.444–2.184	92.7	0.857–3.680
Source category: Algal	3	1.750	0.973–3.149	94.7	Not emphasized (*k* = 3)
Exclude RPT‐0165 small‐n first‐stage screen	30	1.671	1.518–1.840	95.4	1.020–2.737
RPT‐0037 turnip‐only source effect	31	1.698	1.533–1.882	95.3	0.996–2.897
RPT‐0037 maca‐only source effect	31	1.714	1.544–1.902	95.4	0.996–2.947
3‐report legacy add‐back sensitivity	34	1.710	1.556–1.880	94.9	1.025–2.854

*Note:* The primary strict synthesis contains 31 independent StudyIDs. Broad source strata are post hoc exploratory and assigned at the StudyID/intervention level. The three‐report purity‐boundary add‐back is a targeted legacy reclassification sensitivity analysis, not an all‐17 boundary‐study meta‐analysis.

Abbreviations: CI, confidence interval; *I* ^2^, percentage of observed variance attributable to between‐study heterogeneity; *k*, number of independent Study*I*Ds; RoM, response ratio.

All 31 point estimates were above 1, and the lower limit of each conventional normal‐approximation study‐level 95% confidence interval was also above 1. StudyID RoM values ranged from 1.121 to 3.443. This directional uniformity is reported as a feature of the evidence base; it is not interpreted as proof of publication bias.

The 95% prediction interval was 0.999–2.917 and included the null value of 1.0. The pooled mean therefore supports longer endurance on average, while a future comparable experiment could plausibly show little or no benefit.

### 3.5 Sensitivity and Influence Analyses

Sensitivity analyses generally preserved the direction of the primary estimate. The leave‐one‐out analysis is shown in Figure S1. Excluding the five StudyIDs with at least one HIGH SYRCLE domain gave *k* = 26 and RoM 1.721 (Hartung–Knapp 95% CI 1.532–1.934; *I* ^2^ = 95.9%; prediction interval 0.979–3.026). The ST‐MORCHELLA‐FRACTION‐SCREEN experiment with *n* = 2 animals per group was influential for the lower prediction bound. The primary analysis included this *n* = 2/group first‐stage fraction screen (StudyID RoM 3.443) and produced a prediction interval of 0.999–2.917; removing it yielded *k* = 30, RoM 1.671 (95% CI 1.518–1.840), and a prediction interval of 1.020–2.737. The pooled average remained positive, but the statement that a future comparable study could show no benefit was partly sensitive to inclusion of this extreme screening design. The *k* = 34 targeted legacy reclassification analysis restored RPT‐0002, RPT‐0044, and RPT‐0052; it was not an all‐17 boundary‐stratum synthesis.

Egger regression did not show a clear asymmetry signal (*p* = 0.844), but the result was not used to rule out publication or selective‐reporting bias. The corresponding funnel plot is shown in Figure S2. Additional sensitivity analyses are presented in Figure S3. Post hoc source‐stratum estimates were RoM 1.652 (95% CI 1.438–1.897; *k* = 15; *I* ^2^ = 96.8%) for plant/other, 1.776 (1.444–2.184; *k* = 13; *I* ^2^ = 92.7%) for fungal, and 1.750 (0.973–3.149; *k* = 3; *I* ^2^ = 94.7%) for algal sources. No formal test for differences between source strata was performed; these post hoc estimates are descriptive and do not establish superiority of a source category. The persistently high heterogeneity within each stratum indicates that broad source category alone does not account for the variation. Descriptive nominal dose patterns were not interpreted as cross‐study dose–response evidence because dose units, preparation chemistry, and uncertainty differed across studies.

### 3.6 Biochemical Outcomes

Quantitative biochemical synthesis was more limited than the endurance analysis because tissue, unit, sampling time, and exercise exposure varied substantially. Table S5 contains 19 biochemical records suitable for effect‐size calculation from seven report IDs. The recoverable records were concentrated in liver or muscle glycogen, blood lactate, blood urea nitrogen (BUN), blood glucose, lactate dehydrogenase (LDH), creatine kinase (CK), and hemoglobin (Hb), rather than providing uniform coverage of antioxidant, mitochondrial, neurochemical, inflammatory, or microbiota pathways.

Same‐session post‐exhaustion blood lactate could be combined in only three studies and was treated as exploratory (benefit‐oriented Hedges' *g* 3.200; Hartung–Knapp 95% CI −1.435 to 7.835; *I* ^2^ = 92.8%). The corresponding forest plot is shown in Figure S4. Because blood was collected after an exhaustion endpoint that was itself affected by treatment, these values are post‐performance biochemical correlates obtained after different exercise exposures; they do not show that lower lactate caused the endurance effect. Same‐session BUN and blood glucose each had only two compatible studies and were not formally pooled. Glycogen outcomes remained at study level because fixed‐duration and post‐exhaustion sampling schedules differed materially.

The 12‐h post‐exercise values from the Tuber indicum study were kept separate from immediate post‐exhaustion measurements. Viili BUN and lactate values were also kept outside pooled analyses because the source unit requires verification and was preserved as reported. These timing and measurement constraints prevent a statistically significant endurance effect from being equated with one validated biochemical mechanism.

Figure 4 integrates the source/fraction chemistry, biochemical response domains, functional endurance signal, and remaining evidence gaps. It emphasizes that chemical/process characterization and standardized biochemical sampling are prerequisites for translating the average endurance signal into a food‐biochemistry model.

### 3.7 Structured Synthesis of Selected Biochemical Outcomes

Table S13 distinguishes quantitative records suitable for effect‐size calculation (*Q*), reported biochemical findings that could not be pooled (R), and outcomes for which data could not be recovered (NR). Recovered domains included lactate/BUN‐related evidence in 15/31 StudyIDs, energy‐substrate/glycogen/glucose evidence in 12/31, oxidative‐stress evidence in 9/31, muscle‐injury/LDH/CK/Hb evidence in 5/31, mitochondrial or adenosine monophosphate‐activated protein kinase (AMPK)‐related evidence in 5/31, gut‐microbiota/short‐chain fatty acid (SCFA) evidence in 5/31, neurochemical evidence in 2/31, and inflammatory evidence in 1/31. These counts describe biochemical domains identified in the available reports, not necessarily all outcomes measured in the original reports. Only same‐session blood lactate (three studies) met the threshold for exploratory pooling; the remaining domains were described without cross‐study pooling because sampling and measurements were not comparable.

The biochemical domains are therefore interpreted as patterns observed across studies, not as a demonstrated causal chain. These domains are not mutually exclusive, and a StudyID may contribute to more than one domain. Table S13 also makes missingness visible: a pathway may be biologically plausible and reported in selected studies while still lacking enough comparable measurements for meta‐analysis.

### 3.8 Risk of Bias

For the 23 additionally assessed StudyIDs, the two reviewers agreed directly on 172 of 230 domain judgments (74.8%; Cohen's *κ* = 0.181; Cohen 1960). After reconciliation and integration with the eight StudyIDs from the initial phase, the 31‐StudyID matrix contained 14 LOW judgments (4.5%), 290 UNCLEAR judgments (93.5%), and six HIGH judgments (1.9%) across 310 study‐domain cells; the final consensus matrix is provided in Table S8, and reconciliation details are provided in Table S9. The predominance of UNCLEAR judgments reflects an inability to verify safeguards such as randomization, allocation procedures, housing randomization, and blinding; it should not be taken as evidence of low risk. Excluding the five StudyIDs with at least one HIGH domain did not change the direction of the pooled estimate, but that sensitivity analysis cannot resolve the uncertainty represented by the 290 UNCLEAR cells. Figure 3 summarizes the final domain‐level judgments; a supplementary domain‐level summary is provided in Figure S5.

**FIGURE 3:**
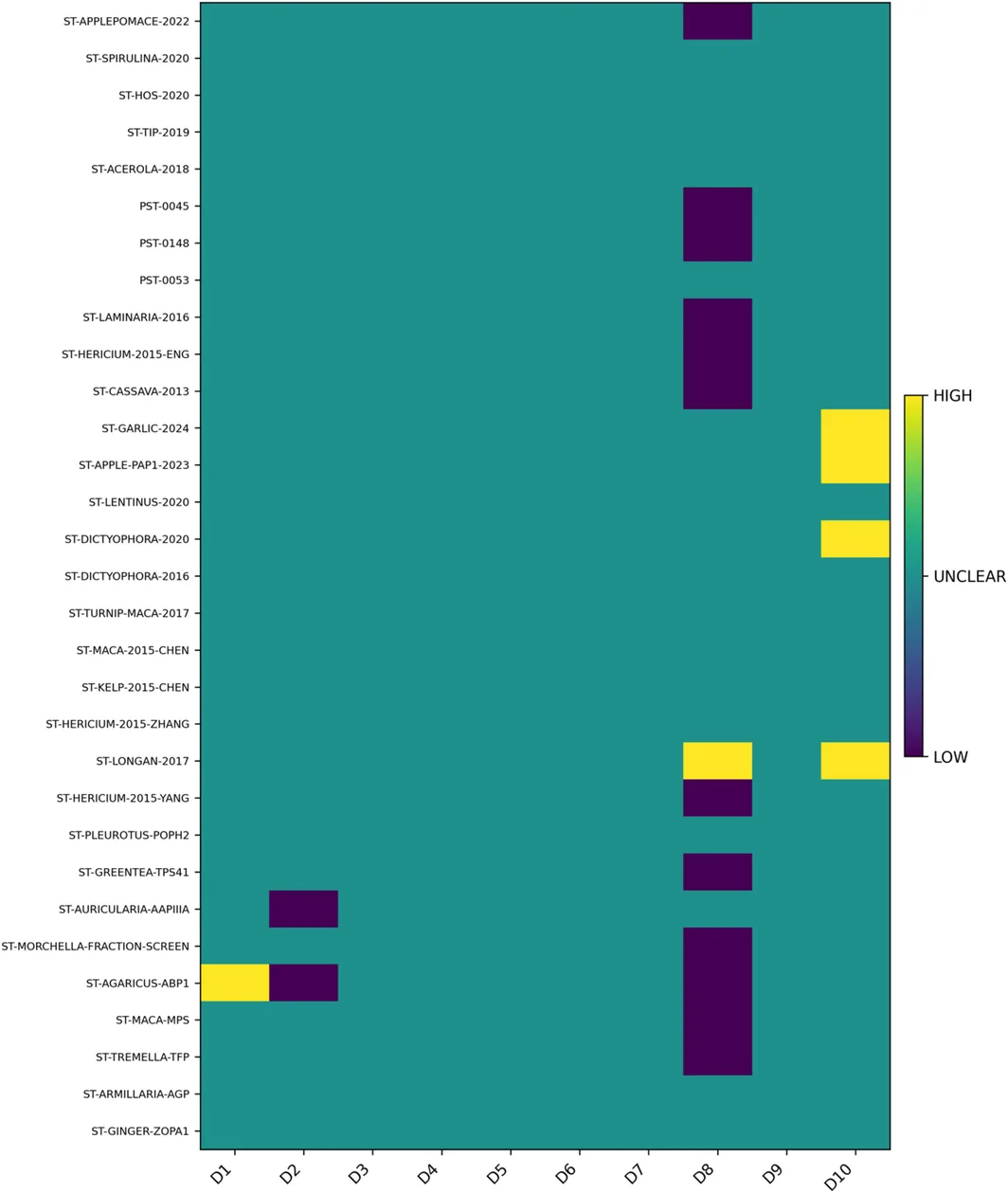
SYRCLE risk‐of‐bias judgments for the 31 StudyIDs in the strict‐primary analysis. Domains are D1 sequence generation; D2 baseline characteristics; D3 allocation concealment; D4 random housing; D5 blinding of caregivers/investigators; D6 random outcome assessment; D7 blinding of outcome assessor; D8 incomplete outcome data; D9 selective outcome reporting; and D10 other sources of bias. Across 310 judgments, 14 were LOW, 290 UNCLEAR, and six HIGH. The predominance of UNCLEAR judgments represents substantial internal‐validity uncertainty rather than evidence of low risk.

## 4 Discussion

### 4.1 Main Finding and Transferability

Across 31 independent StudyIDs, food‐derived polysaccharide supplementation was associated with an average endurance RoM of 1.707. The direction remained positive in the major sensitivity analyses and can be reconstructed from 127 arm‐level records. The size of the average effect, however, should not be confused with a guaranteed effect in another experiment. Heterogeneity was 95.4%, and the primary prediction interval was 0.999–2.917. Earlier reviews mapped natural or dietary polysaccharide sources, doses, models, structural features, and proposed mechanisms (Lei et al. 2024; Liu et al. 2024). The present analysis adds a quantitative distinction between average efficacy and between‐study transferability, together with report‐family auditing, shared‐control handling, and traceable arm‐level provenance.

### 4.2 Composition and Reporting Quality of the Evidence Base

The strict evidence base is modest in independent‐study terms but contains substantial arm‐level detail: 31 StudyIDs and 127 primary‐effect arm records. Group *n* ranged from 2 to 20, and protocols extended from an acute 15‐s post‐gavage screen to 7 weeks of supplementation. Information was not consistently available for strain, sex, treatment duration, exhaustion criteria, administration route, and several chemistry/process descriptors. These gaps constrain both biological transportability and assessment of internal validity.

Risk‐of‐bias reporting points in the same direction. Of 310 SYRCLE domain judgments, 93.5% were UNCLEAR and only 4.5% were LOW. ARRIVE 2.0 emphasizes transparent reporting of design, sample size, randomization, blinding, and outcome measures because missing information prevents readers from judging reliability and reproducibility (Percie du Sert et al. 2020). The pooled estimate remained positive after exclusion of the five HIGH‐risk StudyIDs, but this does not replace the unreported safeguards behind the 290 UNCLEAR judgments. Time‐to‐exhaustion results from swimming and treadmill protocols may reflect different physiological constructs and should not be treated as fully interchangeable.

### 4.3 Source Category Does Not Explain the Heterogeneity

Broad biological source was not sufficient to explain the observed heterogeneity. Plant/other, fungal, and algal strata retained *I* ^2^ values of 96.8%, 92.7%, and 94.7%, respectively. A source label should therefore not be treated as a practical ranking variable. *Var*iation within a source category may arise from extraction and purification, molecular size, composition, dose, treatment duration, animal characteristics, exercise loading, and the definition of exhaustion, but only some of these factors were reported well enough for empirical comparison.

For the same reason, sparse post hoc subgroup results were not used to select a “best” source. Table S14 presents the within‐study nominal dose‐pattern audit, including baseline performance, nominal dose series, preparation chemistry, and exercise protocol. Within published experiments, 24/26 comparable nominal dose series showed a non‐decreasing mean pattern. Because dose units, preparation chemistry, shared‐control structures, and uncertainty differed across studies, that ordering does not establish a dose–response relationship.

### 4.4 Food‐Biochemical Interpretation of Chemical and Processing Heterogeneity

Elevating chemical and processing characterization to the Results section changes the interpretation of the review: the pooled endurance signal should be read through the lens of ingredient identity, not merely broad source category. Table S12 shows that biological source/material, fraction or preparation name, extraction and purification, purity, molecular‐weight information, monosaccharide composition, and structural notes were unevenly reported. Chemically specified examples such as okra stem pectin, *Tuber indicum* polysaccharide, acerola ACWS pectin, purified oat beta‐glucan, garlic GP, apple‐pomace PAP‐1, green‐tea TPS4‐1, Armillaria AGP, and ginger ZOPA‐1 are informative but not interchangeable preparations (Klosterhoff et al. 2018; Li et al. 2020, 2024; Xu et al. 2013; Zhong et al. 2019; Huang et al. 2024; Jing et al. 2024; Sun 2023; Zhou et al. 2025).

The sparse characterization coverage prevents a credible structure–activity meta‐regression or an optimal chemistry/process specification for anti‐fatigue product development. Future studies should therefore treat source, extraction, purification, purity/content, molecular‐weight distribution, monosaccharide profile, linkage/branching information, dose, and food‐matrix delivery as planned experimental variables rather than after‐the‐fact descriptors. This is the key food‐biochemistry implication of the present evidence base.

### 4.5 Biochemical Evidence

The recurring biochemical signals are concentrated in energy‐substrate and fatigue‐metabolite domains, but the biochemical evidence is less reproducible than the primary endurance dataset. Only 19 records from seven report IDs were suitable for effect‐size calculation in Table S5, and only six of those reports map to the STRICT_PRIMARY set. Table S13 therefore lists reported biochemical observations that could not be pooled. These observations may guide future studies, but they do not establish a validated causal chain from polysaccharide chemistry to biochemical response to endurance outcome. Figure 4 summarizes this integrated interpretation of chemistry, biochemical responses, functional outcomes, and evidence gaps.

**FIGURE 4:**
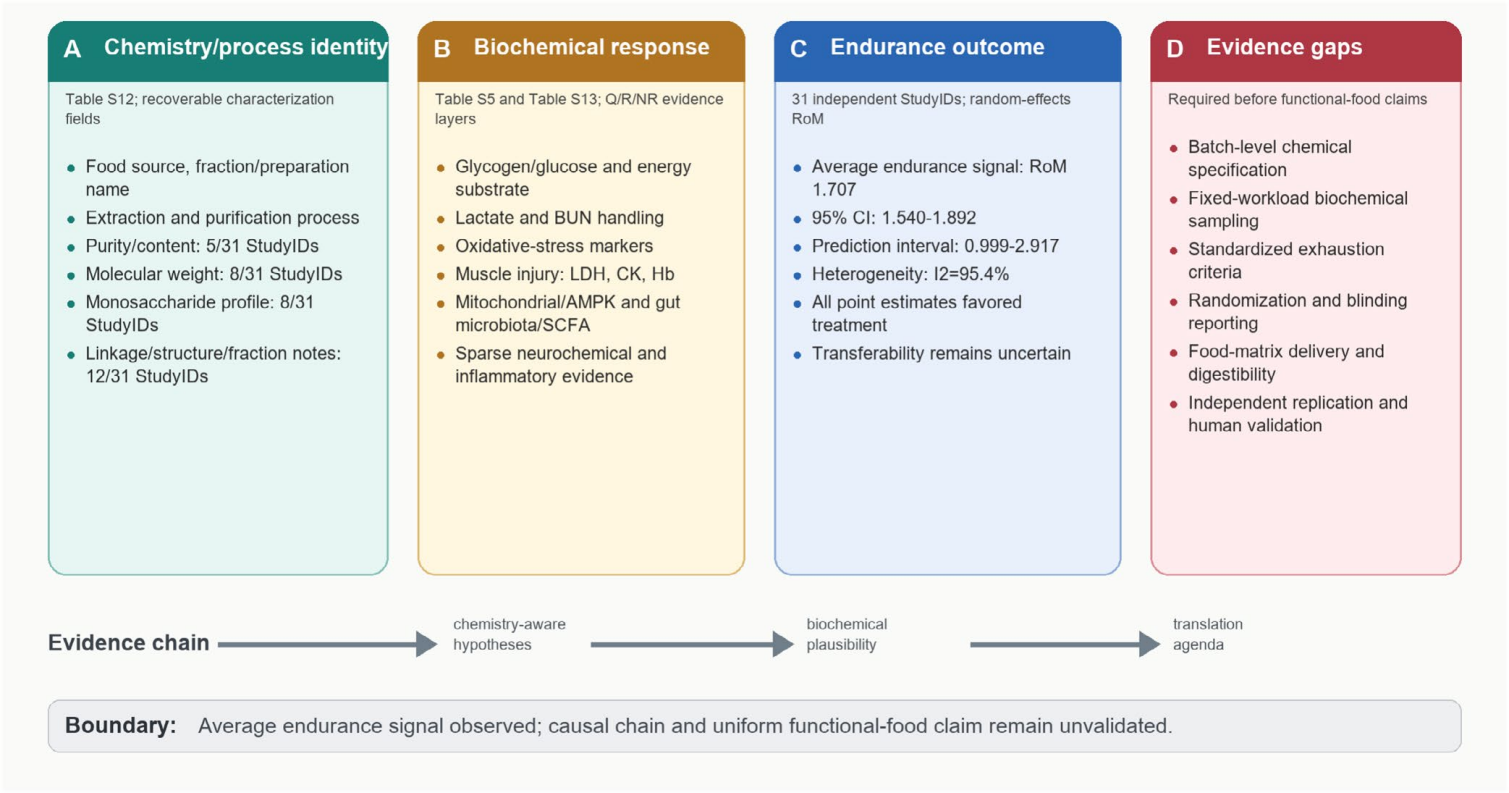
Integrated food‐biochemistry synthesis linking polysaccharide chemistry/process characterization, biochemical response domains, endurance outcomes, and evidence gaps. The figure is interpretive and does not imply a validated causal chain or a uniform functional‐food claim.

### 4.6 Evidence Asymmetry, Influence, and Reproducibility

The study‐level evidence is unusually uniform: every point estimate favored polysaccharide treatment and every conventional study‐level 95% confidence interval was above the null. This pattern does not establish selective publication. The exploratory Egger test was also nonsignificant (*p* = 0.844). Even so, the uniform direction warrants caution because selective outcome reporting, preferential publication of positive animal experiments, or both could contribute. In a highly heterogeneous dataset of 31 studies, Egger regression offers only limited reassurance and should not override the observed asymmetry of the evidence spectrum.

Influence analysis also affects the interpretation of the prediction interval. ST‐MORCHELLA‐FRACTION‐SCREEN used *n* = 2/group across one control and ten first‐stage fractions and produced the largest StudyID effect (RoM 3.443). Removing this experiment left the pooled average positive but changed the prediction interval from 0.999–2.917 to 1.020–2.737. The specific statement that a future comparable study could show little or no benefit is therefore somewhat sensitive to whether this extreme screening design is considered representative. That sensitivity, together with 93.5% UNCLEAR risk‐of‐bias judgments and incomplete chemical and experimental reporting, gives a more informative picture than a simple claim that the pooled result is “robust.”

### 4.7 Implications for Functional‐Food Development

The findings support further evaluation of food‐derived polysaccharides as candidate functional‐food bioactives, but they do not justify a standardized anti‐fatigue claim. Among 26 StudyIDs with at least two comparable ordered dose levels or series, 24 showed a non‐decreasing mean pattern within the published experiment. Because dose units, fractions, preparation chemistry, food matrices, and animal protocols differed across studies, dose optimization should be pursued with chemically defined preparations, prespecified plateau and tolerability criteria, and independent replication rather than inferred from pooled nominal ordering.

Translation also depends on food‐relevant delivery. Administration route was recoverable for only 7/31 StudyIDs (22.6%), and verified routes were mainly gavage/intragastric or direct oral administration rather than incorporation into a conventional food matrix. Product development therefore requires batch‐level composition and purity, molecular‐weight distribution, reproducible extraction, gastrointestinal stability and digestibility, palatability, realistic intake, and achievable exposure after digestion. Safety and tolerability, workload‐relevant outcomes, independent replication, and ultimately human validation are also needed. The current animal evidence supports prioritization, not product readiness.

## 5 Conclusions

Food‐derived polysaccharides were associated with higher average exercise‐endurance capacity in animal fatigue models (RoM 1.707), but the evidence does not support a uniform class effect. Transferability is limited by very high heterogeneity, a primary prediction interval that includes the null, incomplete experimental and chemical/process reporting, an unusually all‐positive study‐level evidence spectrum, and predominantly UNCLEAR risk‐of‐bias judgments. Biochemical observations recur most often in energy‐substrate/glycogen, lactate, and BUN handling, oxidative‐stress, and metabolic domains, whereas pathway‐level mechanisms remain sparse and heterogeneous.

Future studies should standardize exhaustion criteria and fixed‐workload biochemical sampling; report randomization, blinding, attrition, endpoint‐specific *n*, and food‐matrix delivery explicitly; and characterize polysaccharides by biological source, fraction/preparation name, extraction/purification, purity, molecular‐weight distribution, monosaccharide composition, and structural information. Dose‐series experiments should report all arms without treating nominal mean ordering as proof of dose response. Gastrointestinal stability, digestibility, tolerability, and safety should be established before animal efficacy signals are translated into functional‐food claims, followed by independent replication and human studies.

## Author Contributions

**Yingxi Zhao:** conceptualization, writing – review and editing, writing – original draft. **Tianle Zhang:** writing – review and editing, writing – original draft, conceptualization. **Zixin Wang:** investigation, methodology, software. **Xin Huang:** investigation, methodology, software. **Jiazhou Li:** visualization, project administration, resources. **Lijuan Zou:** conceptualization, writing – review and editing, writing – original draft, resources, data curation. **Zheng Li:** writing – review and editing, writing – original draft, resources, data curation, conceptualization.

## Funding

This work was supported by the High‐Level Fund Cultivation Program for Natural Sciences of Guangdong Food and Drug Vocational College (grant no. 2025ZR05) and the Characteristic Innovation Project for General Higher Education Institutions of the Department of Education of Guangdong Province (grant no. 2025KTSCX303).

## Ethics Statement

This systematic review and meta‐analysis used data from previously published animal studies and did not involve new experiments on animals or humans.

## Conflicts of Interest

The authors declare no conflicts of interest.

## Supporting information


**Figure S1:** Leave‐one‐out analysis.
**Figure S2:** Exploratory funnel plot (Egger *p* = 0.844).
**Figure S3:** Robustness sensitivity analyses.
**Figure S4:** Same‐session post‐exhaustion blood lactate.
**Figure S5:** SYRCLE domain‐level summary (31 StudyIDs).

**Table S1:** Search provenance and final rerun/reconstruction control.
**Table S2:** Reports excluded after eligibility assessment and standardized primary reasons.
**Table S3:** Eligible 587‐report corpus: final evidence class, operational 587‐to‐123 gate, and quantitative‐synthesis disposition.
**Table S4:** Biochemical outcome sampling strata and final pooling decisions.
**Table S5:** Effect‐ready biochemical outcome records and study‐level effect estimates.
**Table S6:** Synthesis eligibility flow from 587 eligible reports through the 123 quantitative‐candidate audit to 31 strict‐primary StudyIDs.
**Table S7:** Final strict‐primary StudyID characteristics, endurance effect estimates, quantitative provenance, and bibliography mapping.
**Table S8:** Final SYRCLE risk‐of‐bias consensus matrix for the 31 strict‐primary StudyIDs.
**Table S9:** Reviewer‐A/Reviewer‐B SYRCLE disagreements and publication‐facing reconciliation rationale.
**Table S10:** Eligible 587‐report corpus profile.
**Table S11:** Observed control and StudyID‐level active‐summary endurance evidence across 31 strict‐primary StudyIDs.
**Table S12:** Source‐audited biological source/material, fraction/preparation, administration route, processing and chemistry of the 31 strict‐primary StudyIDs.
**Table S13:** Locator‐backed biochemical evidence matrix across the 31 strict‐primary StudyIDs.
**Table S14:** Descriptive within‐study nominal dose‐pattern assessment.

## Data Availability

The data that support the findings of this study are available in the Supporting Information of this article.
