# Supplementary material for: Exercise Endurance Effects of Food‐Derived Polysaccharides in Animal Models of Exercise‐Induced Fatigue: A Systematic Review and Meta‐Analysis
Source: Food Sci Nutr. 2026 Oct 29;14(11):e72529. doi: 10.1002/fsn3.72529 (PMC13720232; doi:10.1002/fsn3.72529)
Supplement: Supplementary file 1 [file FSN3-14-e72529-s002.docx]

**Supplementary Figures**

Exercise Endurance Effects of Food-Derived Polysaccharides in Animal Models of Exercise-Induced Fatigue: A Systematic Review and Meta-Analysis

**Supplementary Figure S1. Leave-one-out analysis.**

**Supplementary Figure S2. Exploratory funnel plot (Egger p = 0.844).**

**Supplementary Figure S3. Robustness sensitivity analyses.**

**Supplementary Figure S4. Same-session post-exhaustion blood lactate.**

**Supplementary Figure S5. SYRCLE domain-level summary (31 StudyIDs).**
